# A complicated cockroach‐ectomy

**DOI:** 10.1002/rcr2.332

**Published:** 2018-05-17

**Authors:** Jaideep Vazirani, Christiaan Yu, Robert Stirling

**Affiliations:** ^1^ Department of Allergy, Immunology and Respiratory Medicine The Alfred Hospital Melbourne Victoria Australia

**Keywords:** Aspiration, bronchoscopy, foreign body

## Abstract

The exact incidence of foreign body aspiration among adults is unknown, and its clinical presentation is vastly divergent. We report the previously undescribed occurrence of cockroach aspiration in an adult, presenting with a “crawling sensation” in his chest. Flexible endobronchial examination revealed a foreign body impacted in the lingula, resembling the Australian cockroach Periplaneta australasiae. Partial extraction via biopsy forceps was performed and complicated by acute hypoxia on disimpaction, requiring a brief period of bag/mask ventilation. Following the offset of procedural sedation, the remaining foreign body was manually expectorated. A total of 24 h post‐procedure, the patient was febrile with positive blood cultures (Micrococcus luteus). We highlight the importance of definitive airway support during endobronchial interventions and raise the question regarding the significance of transient bacteraemia following bronchoscopic manipulation.

## Introduction

Foreign body aspiration (FBA) is a frequent and life‐threatening occurrence in children. The exact incidence of FBA among adults is unknown. Clinical presentation among adults varies from asymptomatic to the classic triad of cough, dyspnoea, and cyanosis. With the advent of smaller‐diameter, flexible bronchoscopes, the management of FBA has evolved into the practice of respiratory physicians. We present a previously undescribed occurrence of cockroach (*Periplaneta australasiae*) aspiration in an adult, followed by flexible bronchoscopic extraction.

## Case Report

A 42‐year‐old man, with a past medical history of paroxysmal atrial fibrillation, presented with chest tightness, shortness of breath, and a “crawling sensation” in his lungs after inhaling a cockroach. The cockroach had initially landed on his hand and was inadvertently aspirated after he gasped with fear on visualizing the insect. Of note, no alcohol ingestion had preceded the event. On initial examination, his heart rate was 110 (regular), blood pressure 130/80, respiratory rate 22, and oxygen saturation 98% on room air. Breath sounds were decreased, with an expiratory wheeze heard on the left. Initial chest radiograph was normal.

**Figure 1:**
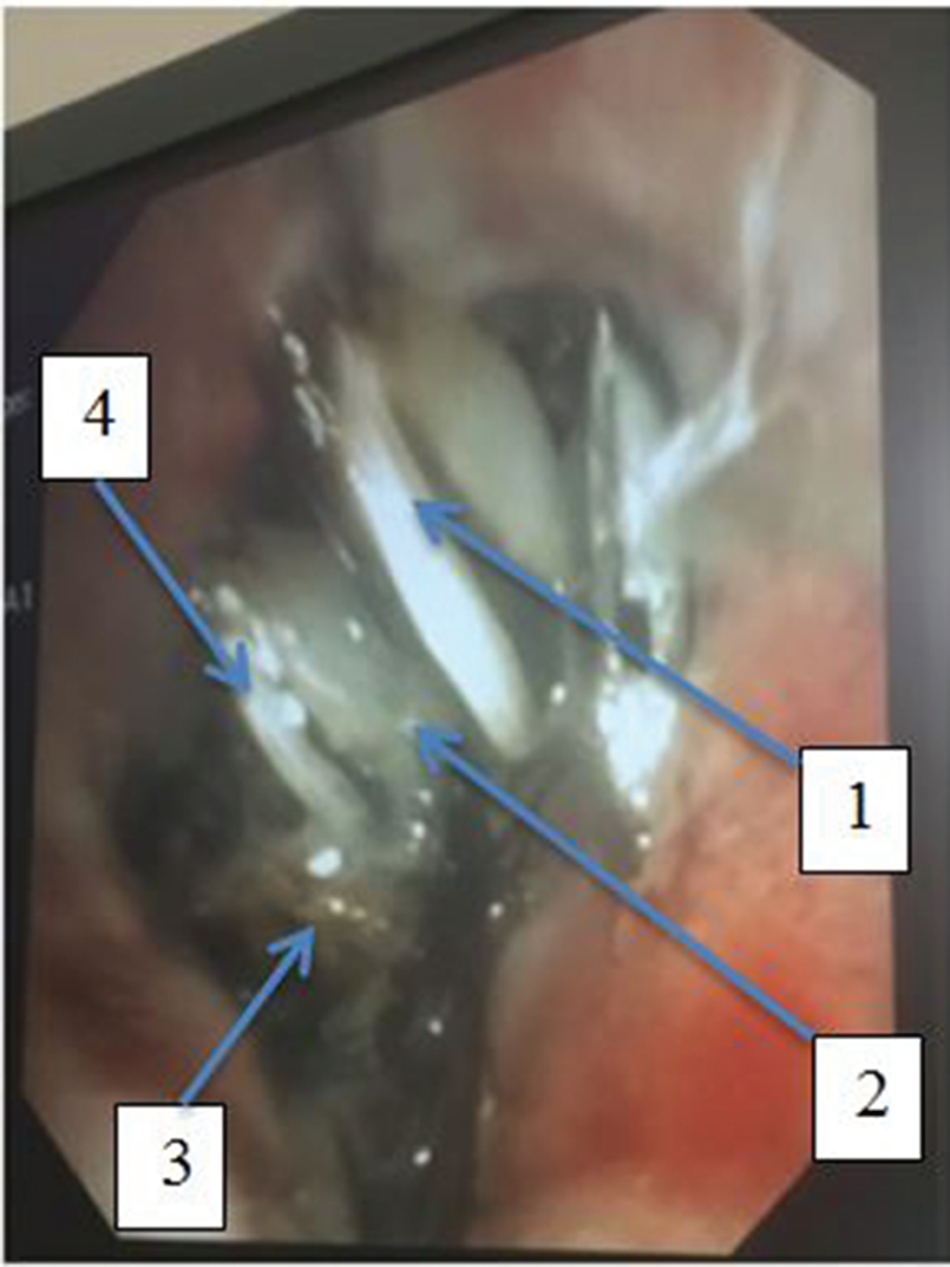
Endobronchial view of cockroach.

A total of 13 h after inhalation, a flexible bronchoscopy was performed and demonstrated the caudal half of the aspirated cockroach (mesothorax, middle legs, abdomen, hind legs, and anal cercus) wedged into the lingula (Fig. 1). There were no other signs of endobronchial irritation. The foreign body was slowly engaged with biopsy forceps and was extracted in part. When a large portion of the cockroach mesothorax was extracted to the trachea, sudden deterioration in oxygen saturation occurred (nadir at 65%), possibly a consequence of the dislodged foreign body with laryngospasm or, alternatively, bronchospasm from cockroach allergen exposure. Bag and mask resuscitation was provided until the patient awoke. On completion of the procedure, the patient coughed up a further segment of the cockroach abdomen. Subsequent rigid bronchoscopy later that day extracted a hind‐wing from the trachea. No further segments of the cockroach were seen.

**Figure 2:**
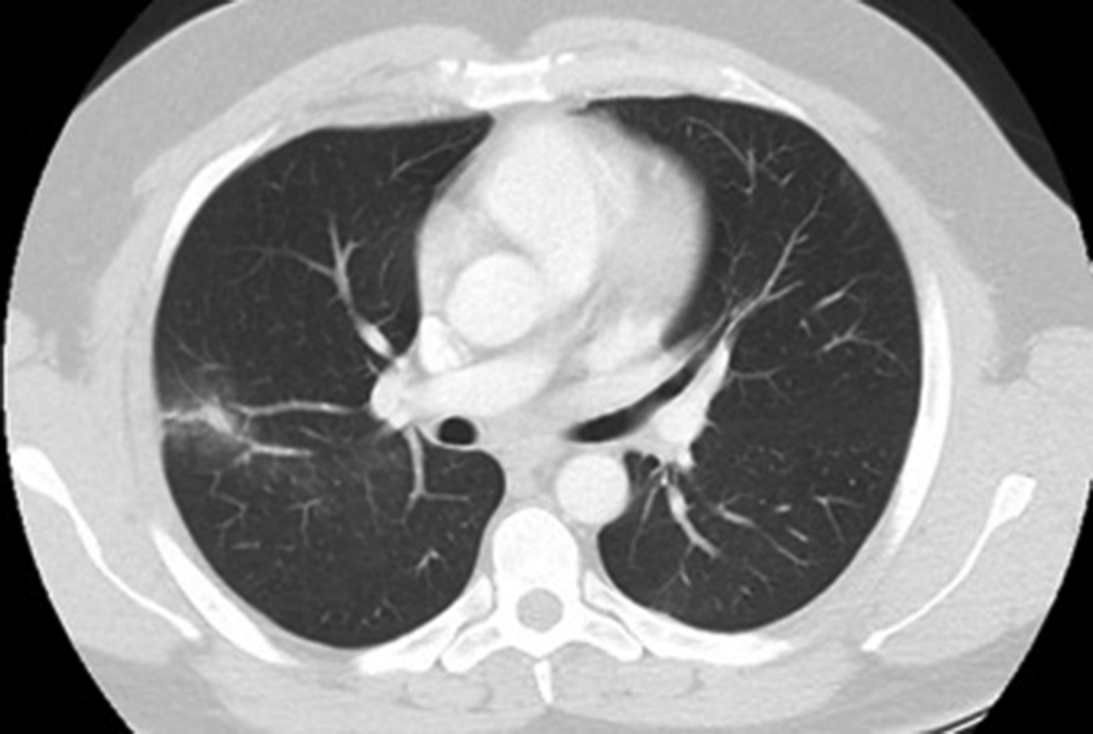
CT chest following extraction of cockroach.

The following day, the patient experienced a fever (38.5 °C), and blood cultures were reported to be positive for *Micrococcus luteus* (*M. luteus*). Antibiotics were commenced, and a repeat chest radiograph and CT chest revealed evolving opacity in the right upper lobe secondary to aspiration pneumonitis, with no evidence of remaining foreign body (Fig. 2).

## Discussion

Foreign body aspiration occurs frequently in children, with most cases occurring in children younger than three years of age. The incidence of FBA in adults is unknown, with one of the largest series quoting 65 FBAs leading to bronchoscopy over 12 years 1.

Risks for FBA among adults have previously been described as older age, abuse of sedative medications, neurological disorders (vascular dementia and Parkinsonism), trauma, tracheostomy, loss of consciousness, and alcoholism 2.

Aspiration of a cockroach has been previously described only in children, with presentations varying from cough and shortness of breath to asphyxiation 3. Even among children, aspiration of an insect is rare and is previously undescribed in an adult.

*Micrococcus luteus* is found in normal flora of the human mouth, mucosae, oropharynx, and upper respiratory tract. *Micrococcus luteus* endocarditis has been described as involving prosthetic heart valves in non‐immunocompromised patients and in native valves in the immunocompromised 4. While not reported following bronchoscopy, transient *M. luteus* bacteraemia has been observed following dental and orthodontic manipulation 5. *Micrococcus luteus* has not previously been linked to cockroaches.

Extraction of FBA from the airway may be complicated by airway obstruction and injury, highlighting the need for this procedure to be carried out by experienced personnel, with facilities available for resuscitation and definitive airway management. The deterioration in our case was likely secondary to laryngospasm and aspiration given the narrow laryngeal aperture seen on withdrawal of the flexible bronchoscope and the changes in right upper lobe seen on post‐bronchoscopy Computerized Tomography. Although an IgE reaction is possible, this was thought to be less likely given the absence of eosinophilia and lack of atopy 6. While a Radioallergosorbent Test for the major cockroach allergen (Blag1) may confirm cockroach sensitization, it would not confirm allergic bronchoprovocation.

This case serves as a reminder that fever following bronchoscopic manipulation can be complicated by transient airway flora bacteraemia, with consequent risks of endocarditis in at‐risk patients.

### Disclosure Statement

Appropriate written informed consent was obtained for the publication of this case report and accompanying images.
